# Downregulation of myostatin pathway in neuromuscular diseases may explain challenges of anti-myostatin therapeutic approaches

**DOI:** 10.1038/s41467-017-01486-4

**Published:** 2017-11-30

**Authors:** Virginie Mariot, Romain Joubert, Christophe Hourdé, Léonard Féasson, Michael Hanna, Francesco Muntoni, Thierry Maisonobe, Laurent Servais, Caroline Bogni, Rozen Le Panse, Olivier Benvensite, Tanya Stojkovic, Pedro M. Machado, Thomas Voit, Ana Buj-Bello, Julie Dumonceaux

**Affiliations:** 1 NIHR Biomedical Research Centre, University College London, Great Ormond Street Institute of Child Health and Great Ormond Street Hospital NHS Trust, 30 Guilford Street, London, WC1N 1EH UK; 2 Genethon, INSERM U951, Univ Evry, Université Paris-Saclay, 91002 Evry, France; 3 Inter-University Laboratory of Human Movement Biology (LIBM)–EA7424 Université Savoie Mont Blanc, Campus Scientifique Technolac, 73376 Le Bourget du Lac Cedex, France; 4 Laboratoire Interuniversitaire de Biologie de la Motricité–EA 7424, Univ Lyon, UJM-Saint-Etienne, Unité de Myologie, Centre Référent Maladies Neuromusculaires Rares Rhône-Alpes, CHU Saint-Etienne, Saint-Étienne, France; 5 Department of Molecular Neuroscience, MRC Centre for Neuromuscular Diseases, Institute of Neurology, University College London, 8-11 Queen Square, London, WC1N 3BG UK; 6 The Dubowitz Neuromuscular Centre, UCL Great Ormond Street Institute of Child Health, 30 Guildford Street, London, WC1N 1EH UK; 7 Groupe Hospitalier Pitié-Salpêtrière Département de Neurophysiologie Clinique, 75651 Paris cedex 13, France; 8 I-Motion–Research Center for Pediatric Neuromuscular Diseases, Armand Trousseau Hospital, Paris, 75571 France; 9 Pediatric Department, Centre Hospitalier Universitaire de Liège, Université de Liège, Liège, 4000 Belgium; 10 Sorbonne Universités, UPMC Univ Paris 06, Centre de Recherche en Myologie, INSERM UMRS974, Institut de Myologie, 105 bd de l’Hôpital, 75013 Paris, France; 11 Centre de référence de Pathologie Neuromusculaire Paris-Est, Institut de Myologie, GHU Pitié-Salpêtrière, Assistance Publique-Hôpitaux de Paris, Paris, 75013 France; 12 Centre for Rheumatology, Division of Medicine, University College London, Rayne Building 4th Floor, 5 University Street, London, WC1E 6JF UK; 13 Present Address: Unité Physiologie et Pathologie Moléculaires des Rétrovirus Endogènes et Infectieux, CNRS UMR 9196, Institut Gustave Roussy, Villejuif, 94805 France

## Abstract

Muscular dystrophies are characterized by weakness and wasting of skeletal muscle tissues. Several drugs targeting the myostatin pathway have been used in clinical trials to increase muscle mass and function but most showed limited efficacy. Here we show that the expression of components of the myostatin signaling pathway is downregulated in muscle wasting or atrophying diseases, with a decrease of myostatin and activin receptor, and an increase of the myostatin antagonist, follistatin. We also provide in vivo evidence in the congenital myotubular myopathy mouse model (knock-out for the myotubularin coding gene *Mtm1*) that a down-regulated myostatin pathway can be reactivated by correcting the underlying gene defect. Our data may explain the poor clinical efficacy of anti-myostatin approaches in several of the clinical studies and the apparent contradictory results in mice regarding the efficacy of anti-myostatin approaches and may inform patient selection and stratification for future trials.

## Abstract (web summary)

Drugs targeting myostatin reverse muscle wasting in animal models, but have limited efficacy in patients. The authors show that the myostatin pathway is downregulated in patients, possibly explaining the poor outcome of anti-myostatin approaches, and that it can be reactivated by correcting disease-causing mutations in mice.

## Introduction

Skeletal muscle mass is controlled by different pathways among them myostatin, which is a member of the transforming growth factor-beta (TGF-beta) family of proteins whose function appears to be conserved across species^1^. Because several spontaneous mutations in the myostatin gene have been correlated with muscle hypertrophy in animals (for review see ref. ^2^) or even in human^3^, myostatin inhibition had been seen as a promising tool to fight muscle atrophy in different diseases including muscle diseases.

Myostatin is a secreted protein, synthetized by skeletal muscle as a precursor which undergoes maturation steps^4, 5^. Several myostatin inhibitory drugs have been designed targeting different stages of the myostatin biosynthesis or pathway among them (i) monoclonal antibodies targeting myostatin, (ii) monoclonal antibodies targeting myostatin’s receptor AcvRII, (iii) AcvRII decoys, (iv) follistatin overexpression which functions as a myostatin antagonist by preventing receptor binding (for review see ref. ^6^). During the last 15 years, at least six molecules (MYO-029, BMS-986089, PF-06252616, ACE-083/-031, BYM338, FS-344) have been developed by pharmaceutical companies to block myostatin pathways (https://clinicaltrials.gov). These molecules are/were evaluated in several neuromuscular diseases that show muscular wasting or atrophy but so far the published results were largely disappointing. Significant improvements in muscle strength or physical function have not been reached^7–9^ with the exception of two small open-label studies using an AAV vector encoding the follistatin isoform FS344 intramuscularly injected in Becker Muscular Dystrophy (BMD, *n* = 6) patients and in Inclusion Body Myositis (IBM, *n* = 6)^10–12^ and 1 small randomised controlled trial using a monoclonal antibody against the AcvRII receptor in IBM patients (*n* = 11 active, 3 placebo)^13^. Several explanations have been proposed, among them the specificity of the drugs themselves and the possibility that they do not target the correct form of myostatin or target other growth factors besides myostatin implicated in muscle mass regulation. However, in animals, several laboratories including ours have demonstrated that myostatin pathway inhibition leads to muscle hypertrophy and enhances tetanic force in controls or in several murine models of muscle diseases such as the *mdx* mouse, a murine model for Duchenne Muscular Dystrophy (DMD)^14, 15^.

We hypothesized that another possible explanation for the poor clinical efficacy of anti-myostatin molecules in several of the human studies was that the expression level of the targeted protein itself was reduced. Indeed, one can easily imagine that a treatment targeting circulating myostatin may not work if the level of circulating myostatin is already very low in patients. So far, only a few articles have described the expression levels of circulating myostatin in patients^16–18^. In our study, the expression levels of different actors of the myostatin network were analyzed at messenger RNA (mRNA) and/or protein levels in the sera and/or biopsies of patients with different muscular diseases and in a mouse model of congenital myotubular myopathy. Our data show that in several neuromuscular diseases the myostatin pathway is shut down at mRNA level in muscle biopsies, leading to low levels of circulating and endogenous muscle myostatin and high-levels of follistatin. The regulation of the myostatin network is disease-dependent, the patients affected by the most atrophying disease showing the strongest extinction of the myostatin pathway. Further inhibition of this pathway by an exogenous compound (monoclonal antibody or vector-mediated inhibition) in the presence of strong down-regulation in severely affected muscles may not be an efficient strategy to increase muscle mass, even though this blockage is reversible upon proper treatment of the primary cause of the disease, as exemplified by the myotubular myopathy model. These data may explain the poor clinical efficacy of most anti-myostatin approaches for neuromuscular diseases to date and may affect patient selection and stratification for future trials.

## Results

### Concentration of serum myostatin in neuromuscular diseases

**Fig. 1:**
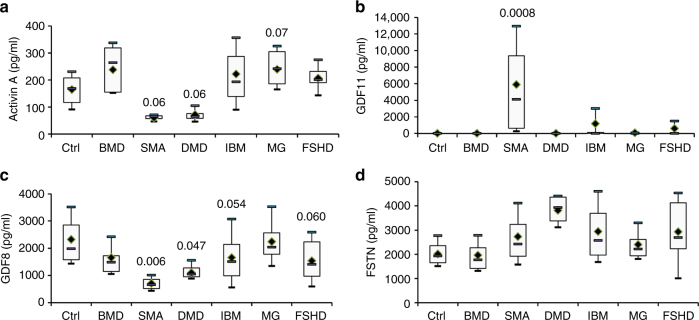
Circulating levels of GDF8, FSTN, ACTIVIN A and GDF11. Circulating levels of either ACTIVIN A **a**, GDF11 **b**, GDF8 **c** and FSTN **d** were measured in healthy control (Ctrl, *N* = 9), Becker Muscular Dystrophy (BMD, *N* = 6), Spinal Muscular Atrophy (SMA, *N* = 4), Duchenne Muscular Dystrophy (DMD, *N* = 4), Inclusion Body Myositis (IBM, *N* = 54), Myastenia Gravis (MG, *N* = 12) or Facioscapulohumeral Dystrophy (FSHD, *N* = 13) patients. Horizontal lines are medians, the extremities of the boxes are delimitated by the first and third quartile, and the whiskers correspond to the 10th and 90th percentile. A one-way ANOVA, followed by the Fisher’s Least Significant Difference multiple comparison test was performed. Degree of freedom = 6

Serum concentrations of myostatin (MSTN or GDF8), follistatin (FSTN), GDF11 and ACTIVIN A were determined in patients affected by several neuromuscular diseases with various levels of muscle atrophy and in controls (summarized in Table 1 and Supplementary Table 1). BMD and DMD share similar clinical signs and symptoms including muscle weakness and atrophy but in BMD, symptoms are milder and patients have a later onset^19^. Both DMD and BMD are caused by different mutations in the *DMD* gene but mutations in DMD patients lead to an absence of any functional dystrophin protein whereas mutations in BMD patients lead to a less functional protein. Spinal Muscular Atrophy (SMA) is characterized by a loss of motor neurons leading to muscle wasting often leading to premature death^20^. Inclusion-Body Myositis (IBM) is the most common age-related muscle disease in the elderly and characterized by a slowly progressive inflammatory and degenerative myopathy resulting in chronic muscle weakness and atrophy^21^. Facioscapulohumeral Dystrophy (FSHD) is the most common muscular dystrophy in adults characterized by the selective atrophy of groups of muscles^22^. Finally, Myasthenia Gravis (MG) is the most common primary disorder of neuromuscular transmission, caused by antibodies to the acetylcholine receptor leading to muscle weakness usually without severe muscle atrophy^23^. For ACTIVIN A, a trend to a lower expression in SMA and DMD patients (*p* = 0.06 for both, one-way ANOVA test, followed by the Fisher’s Least Significant Difference multiple comparison test) was noted (Fig. 1a). No modification of GDF11 was noted, except in SMA sera in which a massive overexpression was observed (Fig. 1b). Concerning GDF8, in the most atrophic (SMA) and most wasting (DMD) muscle diseases studied a two-fold or higher decrease of circulating GDF8 was observed (SMA 30.6% ± 13.7 and DMD 50.6% ± 17.18 GDF8 compared with controls, respectively) (Fig. 1c). Associated with this GDF8 decrease, an important trend to an increase of circulating FSTN was observed (SMA 135.6% ± 71.3 and DMD 189.4% ± 35.2 compared with controls, respectively) (Fig. 1d). BMD, IBM, and FSHD patients, who clinically show a less pronounced muscle atrophy, have higher circulating myostatin than DMD and SMA patients but less than controls (BMD 71% ± 23.7, IBM 71% ± 55.6 and FSHD 66% ± 35 compared to controls, respectively). The levels of circulating FSTN were not increased in BMD patients (85.5% ± 22.4) whereas a trend to an increase was observed in both IBM and FSHD patients (146.3 ± 70.9 and 145.8 ± 72.8). In MG patients, who do not show any atrophy, no modification of GDF8 nor FSTN was observed. Importantly, regarding all these effectors of the myostatin pathway, an important variation across samples is observed in IBM and FSHD patients, suggesting that the myostatin pathway may be significantly down-regulated in some patients whereas there is still preserved myostatin expression in others. A correlation test was performed between GDF8 and FSTN levels, but no correlation was found.

**Table 1:** Characteristics of patients’ sera

		Ctrl (*n* = 9)	BMD (*n* = 6)	SMA (*n* = 4)	DMD (*n* = 5)	IBM (*n* = 54)	MG (*n* = 12)	FSHD (*n* = 13)
Age (years)	Mean	34.2	18.3	11.0	9.2	64.9	46.6	46.8
	Range	23.1–45.3	7.2–48.7	8.9–12.9	6.0–13.9	41.3–87.1	16.5–64.3	14.5–61.2
Gender	Female	6	–	1	–	17	7	4
	Male	3	6	3	5	37	5	9
Age of onset (years)	Mean	–	Early childhood	Infant	Early childhood	56.5	37.3	20
	Range	–	Early childhood	Infant	Early childhood	35–82	14.5–62.3	2–49
Time elapsed (years)	Mean	–	–	–	–	9.5	9.3	11.1
	Range	–	–	–	–	2.7–45.5	2.0–23.9	5.2–13.7

Time elapsed: number of years between the age at evaluation and the age of onset

### Expression of genes implicated in the myostatin pathway

**Fig. 2:**
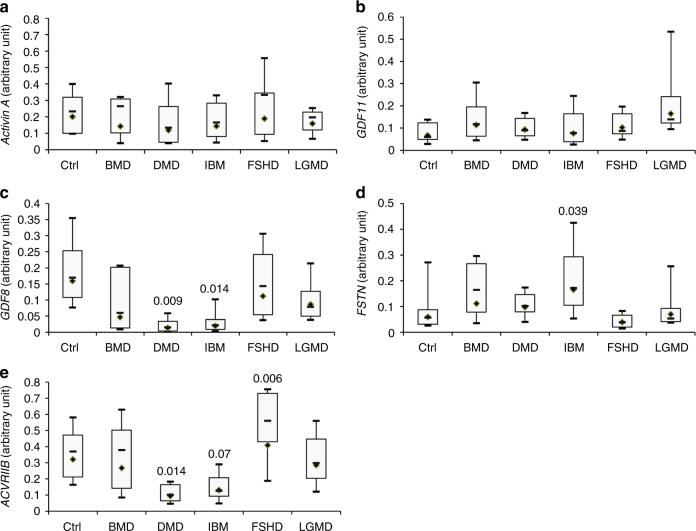
Myostatin pathway in muscle biopsies. mRNA levels of either *ACTIVIN A* **a**, *GDF11* **b**, *GDF8* **c**, *FSTN* **d**, or ACTIVIN A **e** were measured by RT-qPCR in healthy controls (Ctrl, *N* = 9), Becker Muscular Dystrophy (BMD, *N* = 6), Duchenne Muscular Dystrophy (DMD, *N* = 17), Inclusion Body Myositis (IBM, *N* = 17), Facioscapulohumeral Dystrophy (FSHD, *N* = 13) or Limb Girdle Muscular Dystrophy (LGMD, *N* = 11) patients. Horizontal lines are medians, the extremities of the boxes are delimitated by the first and third quartile, and the whiskers correspond to the 10th and 90th percentile. A one-way ANOVA, followed by the Fisher’s Least Significant Difference multiple comparison test was performed. Degree of freedom = 5

As GDF8 is mainly produced by skeletal muscle, the mRNA expression levels of several genes implicated in the myostatin pathway were investigated in muscle biopsies (summarized in Table 2). Unfortunately, SMA skeletal muscle could not be studied as the diagnosis is essentially genetic and a muscle biopsy is not normally performed in SMA patients. No modification of the expression of either *ACTIVIN A* (Fig. 2a) or *GDF11* (Fig. 2b) was observed even if a trend to an overexpression of *GDF11* was noted. A massive down-regulation of *GDF8* was observed in both the DMD and IBM patients as only 8 and 12% of the respective mRNA levels were detectable (Fig. 2c). At the same time, *FSTN* was up-regulated by 2.7 fold in IBM patients (*p* = 0.039) (Fig. 2d). Interestingly, the myostatin receptor *ACVRIIB* was strongly down-regulated in both DMD (30% of residual mRNA, *p* = 0.014) and IBM (40% of residual mRNA, *p* = 0.07) but up-regulated in FSHD (+28%, *p* = 0.006) (Fig. 2e). In Limb-Girdle Muscular Dystrophy (LGMD) and BMD, no significant modification of muscle *GDF8*, *FSTN*, or *ACVRIIB* was observed. An important variability across LGMD and BMD samples was observed but the most atrophying disease (DMD) showed again a general down-regulation of the myostatin pathway. No correlation was found between *GDF8*, *ACVRIIB*, and *FSTN* in all individual diseases. However, concerning FSHD patients, FSHD1 patients express less *GDF8* than FSHD2 patients (*p* = 0.048), but no difference was observed in *FSTN* (Supplementary Fig. 1). FSHD2 patients showed a trend to express more *ACVRIIB* than FSHD1 (+120%, *p* = 0.08). Finally, because fat replacement is not identical in all the diseases or across patients, the expression of *GDF8*, *FSTN*, and *ACVRIIB* were normalized by the expression of the MLC-3 fast myosin heavy chain (encoded by the *MYL1* gene which is expressed in adult in fast muscles^24^), and similar results were obtained (Supplementary Fig. 2).

**Table 2:** Characteristics of patients’ biopsies

		Ctrl (*n* = 9)	BMD (*n* = 6)	DMD (*n* = 17)	IBM (*n* = 17)	FSHD (*n* = 13)	LGMD (*n* = 11)
Age (years)	Mean	43.2	32.5	9.7	70,5	45.0	28.8
	Range	24.0–69.0	1.6–61.3	0.8–15.7	56.9–81.0	13.0–79.4	8.6–57.9
Gender	Female	6	–	–	3	5	5
	Male	3	6	17	14	8	6
Age of onset (years)	Mean	–	23.75	3.3	65.4	27.2	23.3
	Range	–	5–48	2–6	45–78	16–40	4–55
Time elapsed (years)	Female	–	17.7	7	5	21.5	6.7
	Male	–	1.5–36.3	0.7–12.7	0.7–18.5	2.5–38.5	1.9–15.1

Time elapsed: number of years between the age at evaluation and the age of onset

### Myostatin levels impact anti-myostatin approaches

To determine whether or not the endogenous expression level of the myostatin pathway could impact on the success of anti-myostatin approaches, we used the *Mtm1*-KO mouse model. We have chosen the *Mmt1*-KO model because X-linked myotubular myopathy (XLMTM), which is a severe congenital disease due to mutations in the myotubularin coding gene *MTM1*, is characterized by generalized muscle hypotrophy and weakness^25^ and this mouse model recapitulates the muscle atrophy. Moreover, the XLMTM muscle phenotype can be corrected by AAV-mediated gene replacement therapy in the *Mtm1*-KO mouse model of the disease^26, 27^.

**Fig. 3:**
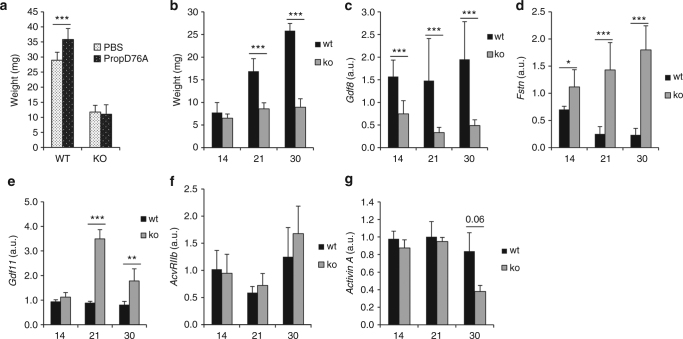
Myostatin network in the tibialis anterior of *Mtm1*-KO mice. The tibialis anterior (TAs) of 3 week-old *Mtm1*-KO mice (KO), or wild type littermates (WT), were injected with either PBS or an AAV vector coding the myostatin pro-peptide D76A mutant (PropD76A) and 2 weeks later, mice were sacrificed and the TAs were weighed **a**. The weights **b**, *Gdf8* **c**, *Fstn* **d**, *Gdf11* **e**, *AcvRIIb* **f**, or *Activin A* **g** mRNAs were measured at 14, 21, or 30 day old in the TAs of either *Mtm1*-KO mice (KO) or wild type littermates (WT) (*n* = 4 and 8 at day 14, 8 and 9 at day 21, 9 and 9 at day 30 for KO and WT mice respectively). All graphs represent mean ± SD, with *p* values calculated by a one-way ANOVA, followed by the Fisher’s Least Significant Difference multiple comparison test

Three week old myotubularin KO (*Mtm1*-KO) and wild type (WT) mice were intramuscularly (in the tibialis anterior (TA)) injected with an AAV coding the myostatin pro-peptide D76A mutant (AAV-PropD76A). While an increase in muscle weight was observed in the WT mice after injection (mean weight = 28.9 ± 0.6 g vs. 35.8 ± 0.9 g for phosphate buffer saline (PBS) and AAV-propD76A injected TA, respectively) *p* = 1.16.10e-10), no muscle growth was observed in the *Mtm1*-KO mice (11.7 ± 0.7 vs. 11 ± 1.8 g, respectively, *p* > 0.05) (Fig. 3a). Because at 3 weeks, *Mtm1*-KO mice already show an important loss of muscle weight of the tibialis anterior compared with WT littermates (6.5 ± 0.9 g vs. 7.8 ± 2.2 at 14 days, 8.6 ± 1.4 g vs. 16.9 ± 2.8 at 21 days and 8.9 ± 1.9 g vs. 25.8 ± 1.6 at 30 days for the *Mtm1*-KO and WT mice respectively, Fig. 3b), the expression level of the myostatin network was investigated. A strong down expression of *Gdf8* (47.6% ± 18.6% of mRNA compared to WT mice at 14 days, 22.5% ± 7.8% at 21 days and 25% ± 6.5% at 30 days, Fig. 3c), associated with a massive up-regulation of *Fstn* (159% ± 44.9% of mRNA compared with WT mice at 14 days, 567% ± 201% at day 21 and 768% ± 190% at day 30, Fig. 3d) and of *Gdf11* (112% ± 18% of mRNA compared with WT mice at day 14, 349% ± 38% at day 21, 178% ± 49% at day 30, Fig. 3e) were observed. *AcvrIIb* and *Activin A* expressions were only barely affected (Fig. 3f, g). These results strongly suggest a correlation between the low expression of expression of *GDF8* and atrophy. This correlation was confirmed by analyzing the weight and *Gdf8* expression in 2 other muscles of the *Mtm1-KO* mouse (Supplementary Fig. 3). In the quadriceps, which is less affected than the TA, there is loss of muscle weight of 25% ± 15% at day 21 and of 46% ± 10% at day 35. The level of *Gdf8* is 71% ± 44 and 38% ± 29% at day 21 and day 35, respectively, compared with the WT animals. Finally, in the least affected muscle, the soleus, there is no muscle loss at day 21, and the myostatin level is unchanged. However, at day 35, the muscle has lost 23% ± 6% of mass and the *Gdf8* level has been reduced by 21% ± 27%. These data suggest that inhibiting the myostatin pathway in an atrophic muscle might not be a successful therapeutic strategy in the *Mtm1*-KO mice at the time point of our analysis because the myostatin pathway is already dramatically down-regulated with 4.4-fold decrease of *Gdf8* mRNA and a 5.6-fold increase of *Fstn* mRNA in the TA of 3 week-old *Mtm1*-KO mice.

**Fig. 4:**
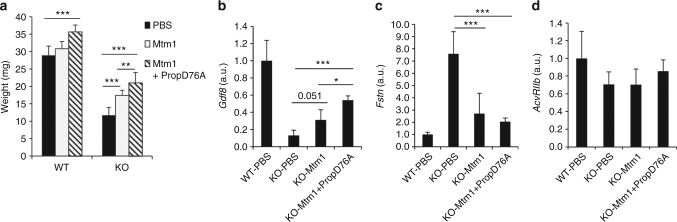
Mtm1 rescue in *Mtm1-KO* mice. The tibialis anterior (TAs) of 3 week-old *Mtm1*-KO mice were injected with either PBS, an AAV vector coding *Mtm1* (Mtm1) or 2 AAVs coding *Mtm1* and propD76A (Mtm1 + PropD76A). Two weeks later, mice were sacrificed and the TAs were weighed **a** and the expression levels of *GDF8* **b**, *Fstn* **c** and *AcvRIIb* **d** mRNAs were measured (*n* = 7 (PBS), 5 (Mtm1), 4 (Mtm1 + PropD76A)). All graphs represent mean ± SD, with *p* values calculated by a one-way ANOVA, followed by the Fisher’s Least Significant Difference multiple comparison test

The tibialis anterior muscles of WT or *Mtm1*-KO mice were next intramuscularly injected with either an AAV coding the *Mtm1* gene (AAV-*Mtm1*) or a combination of the AAV-*Mtm1* gene and the AAV-PropD76A. In the presence of the *Mtm1* protein, muscle histology was greatly improved with an increase of cross-sectional fiber size, and an improved intracellular architecture revealed after NADH-TR staining (Supplementary Fig. 4). The abnormal localizations of the dihydropyrine 1α receptor (DHPR1α) and ryanodine receptor 1 (RYR1) were partially restored. In the *Mtm1*-KO mice, the muscle mass was improved in the presence of the AAV-*Mtm1* (148.1% ± 12.9% of residual mRNA, *p* = 5.3 10e-6 compared with the *Mtm1*-KO injected with PBS) whereas no modification of muscle mass was observed in the WT mice (Fig. 4a). Interestingly, the presence of both AAV-*Mtm1* and AAV-PropD76A allowed a further increase in muscle mass in the *Mtm1*-KO mice (179% ± 25.2%, *p* = 7.15 10e-11, compared with the TAs injected with PBS). A similar effect was observed in the WT mice (123.3% ± 6.8%, *p* = 8.5 10e-9 compared with the WT mice injected with PBS). In conclusion, in both *Mtm1*-KO and WT mice, the simultaneous injection of AAV-PropD76A + AAV-*Mtm1* led to a higher increase in muscle mass than AAV-*Mtm1* alone (Fig. 4a).

To determine if the myostatin pathway was restored by the expression of *Mtm1* in the *Mtm1*-KO mice, the expression levels of *Gdf8*, *Fstn*, and *AcvRIIb* were analyzed in the transduced TAs. An increase of *Gdf8*, associated with a decrease of *Fstn* was observed in the presence of *Mtm1* (Fig. 4b, c), without modification of *AcvRIIb* level (Fig. 4d). The combination of myostatin pathway inhibition and *Mtm1* rescue enhanced both the *Gdf8* increase and the *Fstn* decrease (Fig. 4b, c). These results suggest that the expression of myotubularin in the *Mtm1*-KO mice leading to a 2.4-fold increase of *Gdf8* and a 2.8-fold decrease of *Fstn* was sufficient to enable an anabolic effect of the myostatin propeptide on muscle mass. The muscle atrophy observed in the *Mtm1*-KO mice may not be due to an up-regulation of the myostatin pathway but rather a consequence of the absence of myotubularin.

## Discussion

During the past 12 years at least 15 clinical trials aiming at inhibiting the myostatin pathway have been carried out to improve muscle mass and function in muscular diseases, and several of these studies are still underway (https://clinicaltrials.gov/). Different pathologies were targeted among them BMD, DMD, LGMD, IBM, and FSHD. The concept of anti-myostatin therapy for neuromuscular diseases has been based on the postulate that inhibiting this pathway in patients might lead to an increase in muscle mass and muscle strength/function as it does in normal muscle, which implies that the level of circulating myostatin is high enough to be down-regulated by such a therapeutic approach. However, the results were disappointing: (i) the injection of MYO-29, a recombinant human neutralizing antibody to myostatin, in adult muscular dystrophies (BMD, FSHD, and LGMD) did not improve any of the outcome measures (strength, lean body mass, muscle volume)^8^. (ii) DMD patients treated with ACE-031, a soluble form of activin type IIB receptor, showed a very slight increase in total body lean mass (+4.1% compared with +2.6% in the placebo group) and a non-statistically significant trend for maintenance of 6 min walk test was observed in the ACE-031 treated group, although the study had to be interrupted after 12–16 weeks due to safety concerns^9^. (iii) For sIBM patients treated with bimagrumab, a human monoclonal antibody targeting activin receptors IIA and IIB, a potential benefit in a patient reported outcome (disability scale) was shown, an increased muscle and lean body mass was observed, as well as an improvement in the 6-min walking test in a single dose phase 2 study^13^. However, Novartis has recently announced that, in a late-stage phase2b/3 study, bimagrumab had not met its primary endpoint (6-min walk distance) and did not improve muscle strength (quantitative muscle testing)^28^; (iv) Only one approach in two small phase two open-label studies showed preliminary promising results: in BMD patients, multiple intramuscular injections in three of the four muscles forming the quadriceps of an AAV vector encoding the follistatin isoform FS344 showed an improvement of the 6 min walk test at 6 months post injection^10, 11^. A similar therapeutic approach in IBM patients shown an improvement of the 6 min walk test by 56 m/year (distance annualized to a median 1-year change)^12^. Currently, several clinical trials are underway and results are expected next year.

Different possibilities could explain this absence of functional improvement, among them the drug pharmacokinetics/pharmacodynamics (PK/PD) in the conditions studied so far in humans. A retrospective analysis demonstrated that central clearance of MYO-029 in humans is greater than 2-fold than typical IgG1 mAbs and PK/PD analyses in monkeys suggested that peak and steady state exposures in the MYO-029 trial might achieve only 50 and 10% of the maximum effect seen in monkeys. This would explain why the MYO-029 had a low probability to induce a muscle mass increase in patients^29^. Another explanation could be the lack of specificity of the drugs themselves, GDF8 and GDF11 sharing 90% in their mature region for example. Finally, GDF8 might not be the only ligand implicated in muscle growth to bind ACVRIIB, as it was demonstrated that blocking *AcvRIIb* in *Gdf8* deficient mice further enhances muscle mass^30^. Finally, ACTIVIN A has been described as more prominently regulating muscle mass in primates than does GDF8^31^.

In our study, we have explored another confounding possibility based on the expression levels of circulating and muscle-endogenous proteins implicated in the myostatin pathway. Recently, by using an immunoaffinity (LC–MS/MS) assay, Burch et al.^16^ have published that serum myostatin concentrations are reduced in patients with muscle diseases. They concluded that because myostatin is mainly produced by muscle tissue, these reduced circulating myostatin levels may reflect the net loss of functional muscle mass. Our data however do not support this hypothesis. Indeed, we have observed that whole myostatin pathway is strongly altered in the most atrophying neuromuscular diseases, at both mRNA and protein levels, and that the lower expression of serum myostatin is associated with a reduced muscle expression of *GDF8* mRNA. These results indicated that reduced circulating myostatin levels are not, or at least not only, the reflection of muscle loss but represent an altered myostatin regulation of the diseased tissue. In agreement with this assumption, normalization of myostatin levels against MLC3 did not alter the results, supporting the notion that net muscle loss is not the driver of myostatin reduction. In addition, the muscle atrophy observed in DMD patients is not the consequence of an activation of the myostatin pathway nor to an overexpression of ACTIVIN A. On the contrary, our data indicate that the myostatin pathway, including ACTIVIN A expression, may be intrinsically down-regulated in atrophying or wasting muscle diseases to counterbalance the wasting process. Interestingly, whereas GDF8 and GDF11 are highly homologous, no modification of GDF11 was observed in patient’s sera with the exception of SMA, suggesting that these 2 factors have different biological roles. The down-regulation of the myostatin pathway in human could explain the apparently contradictory results in mice and humans regarding the efficacies of anti-myostatin approaches. Indeed, the outcomes of a clinical trial in DMD patients was not encouraging^9^, while myostatin pathway blockade has been successful in *mdx* mice^14, 15, 32^. Despite the fact that Duchenne patients and *mdx* mice share a mutation in the same gene, no important muscle atrophy is observed before 6 months of age in the *mdx* mouse^33, 34^ and experiments are usually performed before this age. Moreover, even if myostatin levels are lower in *mdx* mouse than in wild-type mouse, the endogenous circulating myostatin level is at least 50 times higher in mice than in humans^16^. This could be one of the reasons why anti-myostatin approaches in the *mdx* model were successful.

Finally, one of the most important questions raised by our work concerns the usefulness of blocking the myostatin pathway in diseases in general and in neuromuscular diseases in particular. Concerning the non-muscular diseases in which the main affected organ is not the muscle (such as cancer), a deep experimental evaluation of the therapeutic potential of the anti-myostatin approaches needs to be performed. Concerning the neuromuscular diseases, in slowly progressive pathologies such as BMD or FSHD, an important variability of both myostatin and follistatin circulating proteins is observed across samples, suggesting that at least some patients may be eligible for an anti-myostatin approach. However, in the most wasting neuromuscular diseases such as DMD, the whole myostatin pathway is down-regulated. Interestingly, in the *Mtm1*-KO mouse model, the restoration of *Mtm1* expression is associated with a normalization of the myostatin pathway, indicating by analogy that at least partial restoration of the dystrophin protein might be necessary before the inhibition of myostatin. Such an assumption is supported by the higher circulating myostatin levels in BMD compared with DMD, and experimentally by the stronger effect of anti-myostatin therapy in *mdx* mice if complemented by dystrophin restoration through exon skipping^14, 32^. Therefore, for future trials of anti-myostatin therapy patient eligibility should be tested by ascertaining sufficient levels of the therapy target and taking into account that general circulating myostatin levels may not be representative of the muscle-intrinsic levels of affected target muscles. Furthermore, in the most atrophying diseases, the mutated gene might need to be rescued first in order to restore myostatin expression before inhibiting the myostatin pathway becomes a therapeutic option.

## Methods

### Patients

The collection of sera and biopsies were approved by the “Comité de Protection des Personnes” Paris VI and the French regulatory agency (ANSM) (CCP#99–12, ID RCB 2012-A01277-36), the research and Development Office (#DN 12DN29), and the East Central London Research Ethics Committee 1 (reference number 10/H0721/28 and 12/LO/1557—Queen Square REC). An informed consent was obtained from all subjects. The characteristics of the patients are described in Tables 1 and 2 and Supplementary Table 1.

### Mouse experiments

Mice were handled according to French and European legislation on animal care and experimentation, and protocols were approved by the institutional ethical committee. The constitutive knock-out of the myotubularin gene (*Mtm1*-KO, also named BS53d4-129pas) was generated previously by homologous recombination^35^. Wild-type littermate males were used as controls. Sample size was chosen based on previous experience in the laboratory with the mouse model and response to treatments. No pre-established inclusion/exclusion criterion was applied. Animal were not randomized for allocation in the experimental groups and animal studies were not performed under a blinding procedure. Only males were used in the experiments.

### Generation of recombinant AAV vector and delivery

A recombinant serotype 1 AAV vector containing mutated myostatin propeptide D76A under the CMV promoter (AAV-PropD76A) was produced, as previously described^36^. Mouse *Mtm1* complementary DNA (cDNA) (AF073996, NCBI) was cloned in the AAV expression pGG2-DES plasmid, which contains the human desmin promoter. Recombinant serotype 1 viral particles (AAV1-*Mtm1*) were obtained by a tri-transfection procedure from HEK293 cells as previously described^26^. Vector titers were expressed as viral genomes per ml (vg/ml).

AAV vectors were intramuscularly delivered to 3 week-old KO male mice and age matched wild type males. Mice were anesthetized by intraperitoneal injection of 5 μL/body gram of ketamine (20 mg/mL, Virbac) and xylazine (0.4%, Rompun, Bayer). TA muscles were injected with 3.5 × 10^9^ vg of AAV-CMV- PropD76A, 5 × 10^9^ vg of AAV-*Mtm1* or sterile PBS solution. Muscles were dissected 14 days after injection and frozen in either liquid nitrogen-cooled isopentane or liquid nitrogen for histological and molecular assays.

### ELISA analysis

Peripheral venous blood was collected from healthy and patients’ volunteers using serum separator tubes (10 mL). After 30 min on the benchcoat at room temperature, the tubes were centrifuged at 2000 rpm for 10 min at 4 °C. The collected serum (5 mL) was aliquoted and stored at −80 °C until further use. The concentrations of either GDF8, FOLLISTATIN, GDF11, or ACTIVIN A in the sera were measured using an ELISA kit (respectively # DGDF8, # DFN00, # DY1958, and # DAC00B R&D Systems Europe, Ltd, Abingdon, United Kingdom) according to the manufacturer’s instructions. The optical density was measured using a microplate reader (Infinite 200 Pro, Tecan Group Ltd., Männedorf, Switzerland). Importantly, the myostatin immunoassay was designed to recognize mature GDF8. According to the manufacturer, no significant cross-reactivity or interference was observed in the presence of 50 ng/ml (20 times more than the highest value measured in our experiment) of different proteins including the GDF8 propeptide, GDF11 or GDF15. We have experimentally confirmed this result by using 4000 pg/ml of recombinant GDF11. The absence of cross reactivity with FOLLISTATIN (8000 pg/ml) and ACTIVIN A (4000 pg/ml) was also experimentally validated.

### RNA extraction and real-time PCR

For murine samples, total RNA was purified from muscles of males using TRIzol reagent (Life Technologies, Saint Aubin, France) according to manufacturer’s instructions. RNA concentration was measured by spectrophotometry (OD 260 nm) using a nanodrop ND-1000 spectrophotometer (Thermo Scientific, Wilmington, DE, USA) and RNA integrity was verified by electrophoresis using ethidium bromide. After DNAse treatment (Ambion), RNA was reverse transcribed using Super Script II RNase H Reverse Transcriptase (Invitrogen) in the presence of Random Primers (Promega). Real-time PCR was performed at 60 °C as melting temperature and with primers described in Supplementary Table 2 using an ABI Prism 7900 apparatus (Applied Biosystems) in a final volume of 25 µl with reverse transcriptase, forward and reverse primers (0.5 nmol/ml) and SYBRGreen Mastermix (Roche, Basel, Switzerland).

For human samples, cryopreserved tissues were transferred in tube containing 1.4 mm ceramic beads (Precellys, Bertin Corp, Maryland, United State) plus 1 mL of Trizol (Life technologies, Saint Aubin, France) and shaked three times at 5700 rpm for 30 s. Between each cycle, tubes were incubated in ice during at least 1 min. Total RNAs were extracted using trizol according to the manufacturer’s protocol (Life technologies, Saint Aubin, France). The quantity of RNA was determined using a nanodrop ND-1000 spectrophotometer (Thermo Scientific, Wilmington, DE, USA). The reverse transcription was described previously and realized on 1 µg of total RNA in a final volume of 10 µl using the transcriptor first strand cDNA synthesis kit (Roche, Meylan, France)^37^. qPCRs were performed on a LightCycler 480 Real-Time PCR System (Roche, Meylan, France) in a final volume of 9 µl with 0.4 µl of reverse transcriptase (RT) product, 0.18 µl each of forward and reverse primers (20 pmol/ml) and 4.5 µl of SYBRGreen Mastermix (Roche, Basel, Switzerland). After qPCR, the PCR products were run on a 2% agarose gel and were cloned using the Topocloning kit (Life Technologies, Saint Aubin, France) and sequenced. Primers used in this study are described in Supplementary Table 1.

Quantitative PCR (qPCR) was designed according to the MIQE standards^38^. Among the 87 items to review, 57 were classified as essential. All were followed. In particular, to determine the best human housekeeping gene, five genes were evaluated: *B2M*, *GAPDH*, *GUS*, *P0*, and *PPIA*. A MANOVA test has demonstrated that none of these genes were suitable for housekeeping since significant statistical changes were observed between the different groups. *GUS*, *P0*, and *PPIA* were then chosen to calculate the expression normalization factor for each sample using geNorm software (V3.5). To determine the best housekeeping mouse gene, 6 genes were evaluated: *Gapdh*, *P0*, *Hprt1*, *β-actin*, *β-tubulin*, and *18 S,* and *P0* was chosen.

### Statistical analysis

A one-way ANOVA was used for all the experiments, followed by the Fisher’s Least Significant Difference multiple comparison test. A sample-size power analysis was not performed. Differences were considered to be statistically different at p* < 0.05; **<0.01; ***<0.001

### Data availability

All relevant data are available from the authors upon reasonable request.

## Electronic supplementary material

Supplementary Information

## References

[1] McPherron AC, Lawler AM, Lee SJ (1997). Regulation of skeletal muscle mass in mice by a new TGF-beta superfamily member. Nature.

[2] Lee SJ (2004). Regulation of muscle mass by myostatin. Annu. Rev. Cell Dev. Biol..

[3] Schuelke M (2004). Myostatin mutation associated with gross muscle hypertrophy in a child. N. Engl. J. Med..

[4] Lee SJ, McPherron AC (2001). Regulation of myostatin activity and muscle growth. Proc. Natl Acad. Sci. USA.

[5] Lee SJ (2010). Extracellular regulation of myostatin: a molecular rheostat for muscle mass. Immunol. Endocr. Metab. Agents Med. Chem..

[6] Cohen S, Nathan JA, Goldberg AL (2015). Muscle wasting in disease: molecular mechanisms and promising therapies. Nat. Rev. Drug. Discov..

[7] Garber K (2016). No longer going to waste. Nat. Biotechnol..

[8] Wagner KR (2008). A phase I/IItrial of MYO-029 in adult subjects with muscular dystrophy. Ann. Neurol..

[9] Campbell C (2016). Myostatin inhibitor ACE-031 treatment of ambulatory boys with Duchenne muscular dystrophy: results of a randomized, placebo-controlled clinical trial. Muscle Nerve.

[10] Al-Zaidy SA (2015). Follistatin gene therapy improves ambulation in becker muscular dystrophy. J. Neuromuscul. Dis..

[11] Mendell JR (2015). A phase 1/2a follistatin gene therapy trial for becker muscular dystrophy. Mol. Ther..

[12] Mendell JR (2017). Follistatin gene therapy for sporadic inclusion body myositis improves functional outcomes. Mol. Ther..

[13] Amato AA (2014). Treatment of sporadic inclusion body myositis with bimagrumab. Neurology.

[14] Dumonceaux J (2010). Combination of myostatin pathway interference and dystrophin rescue enhances tetanic and specific force in dystrophic mdx mice. Mol. Ther..

[15] Bechir N (2016). ActRIIB blockade increases force-generating capacity and preserves energy supply in exercising mdx mouse muscle in vivo. FASEB J..

[16] Burch PM (2017). Reduced serum myostatin concentrations associated with genetic muscle disease progression. J. Neurol..

[17] Anaya-Segura MA (2015). Non-invasive biomarkers for duchenne muscular dystrophy and carrier detection. Molecules.

[18] Awano H (2008). Wide ranges of serum myostatin concentrations in Duchenne muscular dystrophy patients. Clin. Chim. Acta.

[19] Flanigan KM (2014). Duchenne and becker muscular dystrophies. Neurol. Clin..

[20] Farrar MA (2016). Emerging therapies and challenges in spinal muscular atrophy. Ann. Neurol..

[21] Needham M, Mastaglia FL (2016). Sporadic inclusion body myositis: a review of recent clinical advances and current approaches to diagnosis and treatment. Clin. Neurophysiol..

[22] Wang LH, Tawil R (2016). Facioscapulohumeral dystrophy. Curr. Neurol. Neurosci. Rep..

[23] Gilhus NE (2016). Myasthenia gravis. N. Engl. J. Med..

[24] Schiaffino S (2015). Developmental myosins: expression patterns and functional significance. Skelet. Muscle.

[25] Jungbluth H, Wallgren-Pettersson C, Laporte J (2008). Centronuclear (myotubular) myopathy. Orphanet. J. Rare Dis..

[26] Buj-Bello A (2008). AAV-mediated intramuscular delivery of myotubularin corrects the myotubular myopathy phenotype in targeted murine muscle and suggests a function in plasma membrane homeostasis. Hum. Mol. Genet..

[27] Childers MK (2014). Gene therapy prolongs survival and restores function in murine and canine models of myotubular myopathy. Sci. Transl. Med..

[28] Amato, A.A. et al., A randomized, double-blind, placebo-controlled study of bimagrumab in patients with sporadic inclusion body myositis. *Arthritis Rheumatol*. 68 http://acrabstracts.org/abstract/a-randomized-double-blind-placebo-controlled-study-of-bimagrumab-in-patients-with-sporadic-inclusion-body-myositis/ (2016).

[29] Singh P (2016). Translational pharmacokinetic/pharmacodynamic analysis of MYO-029 antibody for muscular dystrophy. Clin. Transl. Sci..

[30] Lee SJ (2005). Regulation of muscle growth by multiple ligands signaling through activin type II receptors. Proc. Natl Acad. Sci. USA.

[31] Latres E (2017). Activin A more prominently regulates muscle mass in primates than does GDF8. Nat. Commun..

[32] Lu-Nguyen NB (2015). Combination antisense treatment for destructive exon skipping of myostatin and open reading frame rescue of dystrophin in neonatal mdx mice. Mol. Ther..

[33] Pastoret C, Sebille A (1993). Time course study of the isometric contractile properties of mdx mouse striated muscles. J. Muscle Res. Cell Motil..

[34] Pastoret C, Sebille A (1995). mdx mice show progressive weakness and muscle deterioration with age. J. Neurol. Sci..

[35] Buj-Bello A (2002). The lipid phosphatase myotubularin is essential for skeletal muscle maintenance but not for myogenesis in mice. Proc. Natl Acad. Sci. USA.

[36] Bartoli M (2007). AAV-mediated delivery of a mutated myostatin propeptide ameliorates calpain 3 but not alpha-sarcoglycan deficiency. Gene. Ther..

[37] Mariot V (2015). Correlation between low FAT1 expression and early affected muscle in facioscapulohumeral muscular dystrophy. Ann. Neurol..

[38] Bustin SA (2009). The MIQE guidelines: minimum information for publication of quantitative real-time PCR experiments. Clin. Chem..

