# Supplementary material for: Downregulation of myostatin pathway in neuromuscular diseases may explain challenges of anti-myostatin therapeutic approaches
Source: Nat Commun. 2017 Nov 30;8:1859. doi: 10.1038/s41467-017-01486-4 (PMC5709430; doi:10.1038/s41467-017-01486-4)
Supplement: Supplementary file 1 — Supplementary Information [file 41467_2017_1486_MOESM1_ESM.pdf]

**Supplementary table 1 :** Characteristics of LGMD's biopsies

| | LGMD2A (n=1) | LGMD2B (n=4) | LGMD2C (n=2) | LGMD2D (n=2) | LGMD2L (n=2) |
|---|---|---|---|---|---|
| **Age (years)** | | | | | |
| Mean | 44,0 | 34,9 | 11,2 | 16,0 | 39,6 |
| Range | - | 24,2 - 50,1 | 8,6 - 13,8 | 12,1 - 20,0 | 21,3 - 57,9 |
| **Gender** | | | | | |
| Female | - | 3 | 1 | 1 | - |
| Male | 1 | 1 | 1 | 1 | 2 |
| **Age of onset (years)** | | | | | |
| Mean | 40,0 | 30,0 | 4,5 | 8,0 | 55,0 |
| Range | - | 25 - 35 | 4 - 5 | 6 - 10 | - |

**Supplementary table 2:** Oligonucleotides used in this study

| Gene symbol | Accession n° | Name | 5' 3' | Amplicon length |
|---|---|---|---|---|
| *Actb* | NM_007393.5 | b-actin_F | CTGGCTCCTAGCACCATGAA | 123 |
| | | b-actin_R | CTGCTTGCTGATCCACATCT | |
| *Activin A* | NM_008380.2 | Activin A -F | CACACTTCTGCACGCTCCAC | 92 |
| | | Activin A -R | TTTGCCGAGTCAGGCACAG | |
| *Tubb5* | NM_011655.5 | b-tubulin_F | CCTTCATTGGAAACAGCACA | 222 |
| | | b-tubulin_R | CCTCCTCTCCGAAATCCTCT | |
| *Gapdh* | NM_001289726.1 | Gapdh_F | TTGTGATGGGTGTGAACCAC | 283 |
| | | Gapdh_R | TTCAGCTCTGGGATGACCTT | |
| *Gdf11* | NM_010272 | Gdf11 - F | ATCAGCCGGGAGGTAGTGAA | 159 |
| | | Gdf11 - R | CTGGGCCATGCTTATGACCGT | |
| *Hprt* | NM_013556.2 | Hprt1_F | GCAAACTTTGCTTTCCCTGG | 85 |
| | | Hprt1_R | ACTTCGAGAGGTCCTTTTCACC | |
| *Rplp0* | NM_007475.5 | P0_F | CTCCAAGCAGATGCAGCAGA | 87 |
| | | P0_R | ATAGCCTTGCGCATCATGG | |
| *Acvr2b* | NM_007397.3 | ActrIIB_F | GCTCAGCTCATGAACGACT | 68 |
| | | ActrIIB_R | CTCTGCCACGACTGCTTGT | |
| *Fst* | NM_001301373.1 | Fstn_F | CTCTTCAAGTGGATGATTTTC | 345 |
| | | Fstn_R | ACAGTAGGCATTATTGGTCTG | |
| *Mstn* | NM_010834.3 | Mstn_F | GCACTGGTATTTGGCAGAGTA | 345 |
| | | Mstn_R | CACACTCTCCTGAGCAGTAAT | |
| *ACTIVIN A* | ENST00000242208.4 | F-ACTIVIN A | TTATGGAGCAGACCTCGGAG | 75 |
| | | R-ACTIVIN A | AAATCTCGAAGTGCAGCGTC | |
| *B2M* | NM_004048 | F_B2M | CTCTCTTTCTGGCCTGGAGG | 67 |
| | | R_B2M | TGCTGGATGACGTGAGTAAACC | |
| *GAPDH* | ENST00000229239 | F-GAPDH2 | AAGGTGAAGGTCGGAGTCAACGG | 199 |
| | | R-GAPDH2 | TGACAAGCTTCCCGTTCTCAGCC | |
| *GUS* | ENST00000304895 | F-GUS | CTCATTTGGAATTTTGCCGATT | 81 |
| | | R-GUS | CCGAGTGAAGATCCCCTTTTTA | |
| *RPLP0* | ENSG00000089157 | F-P0 | TCCAGGCTTTAGGTATCACCAC | 94 |
| | | R-P0 | GCTCCCACTTTGTCTCCAGTC | |
| *PPIA* | ENST00000355968 | F-PPIA | CCTAAAGCATACGGGTCCTG | 133 |
| | | R-PPIA | TTTCACTTTGCCAAACACCA | |
| *ACVR2B* | ENST00000352511 | F52-AcvRIIb | CTCCTCTGGGGATCGCTGT | 84 |
| | | R135-AcvRIIb | CTCCCAGTTGGCGTTGTAGT | |
| *FSTL* | ENST00000256759 | F1-FSTL | CGGCTGAGCACCTCGTG | 155 |
| | | R1-FSTL | TTCTTGTTCATTCGGCATTT | |
| *GDF11* | ENST00000257868.9 | F-GDF11 | ATTGGCAGAGCATCGACTTC | 182 |
| | | R-GDF11 | TTTTGTGTTCTCTAGGACTCG | |
| *MSTN* | NM_005259 | F-972 | TTTTACCCAAAGCTCCTCCA | 258 |
| | | R-3017 | GAGTCTCGACGGGTCTCAAA | |
| *MYL1* | ENST00000352451 | F-MYL1 | GCAATGAAGAGCTGAATGCCA | 126 |
| | | R-MYL1 | TGTCAAAGACACGCAGACCCT | |

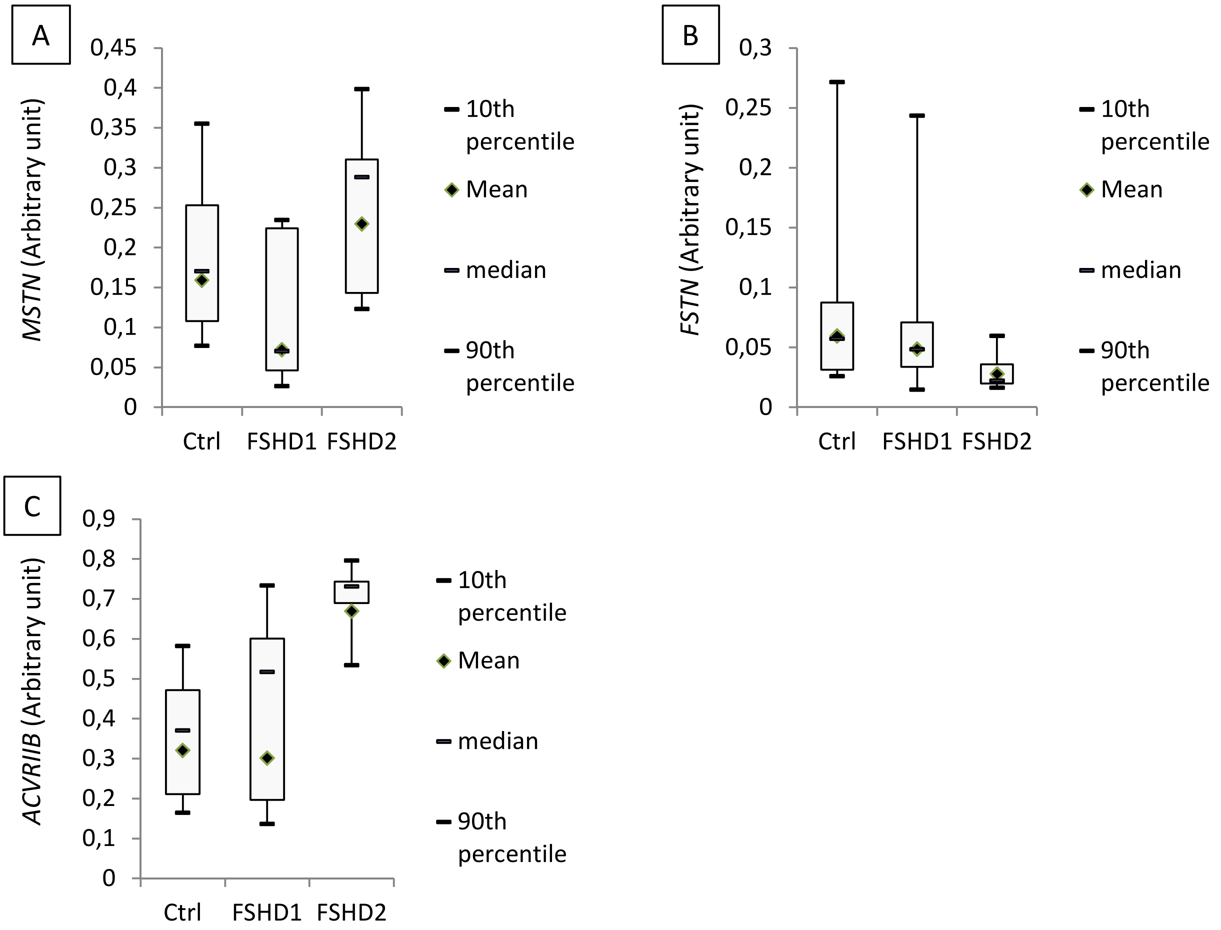


**Supplementary figure 1:** mRNA levels of either *MSTN (A), FSTN (B), or ACVRIIB (C)* were measured by RT-qPCR in in healthy controls (Ctrl, N=9), FSHD1 (N=8), FSHD2 (N=5) patients. Horizontal lines are medians, the extremities of the boxes are delimitated by the first and third quartile, and the whiskers correspond to the 10th and 90th percentile. A one-way ANOVA, followed by the Fisher's Least Significant Difference multiple comparison test was performed.

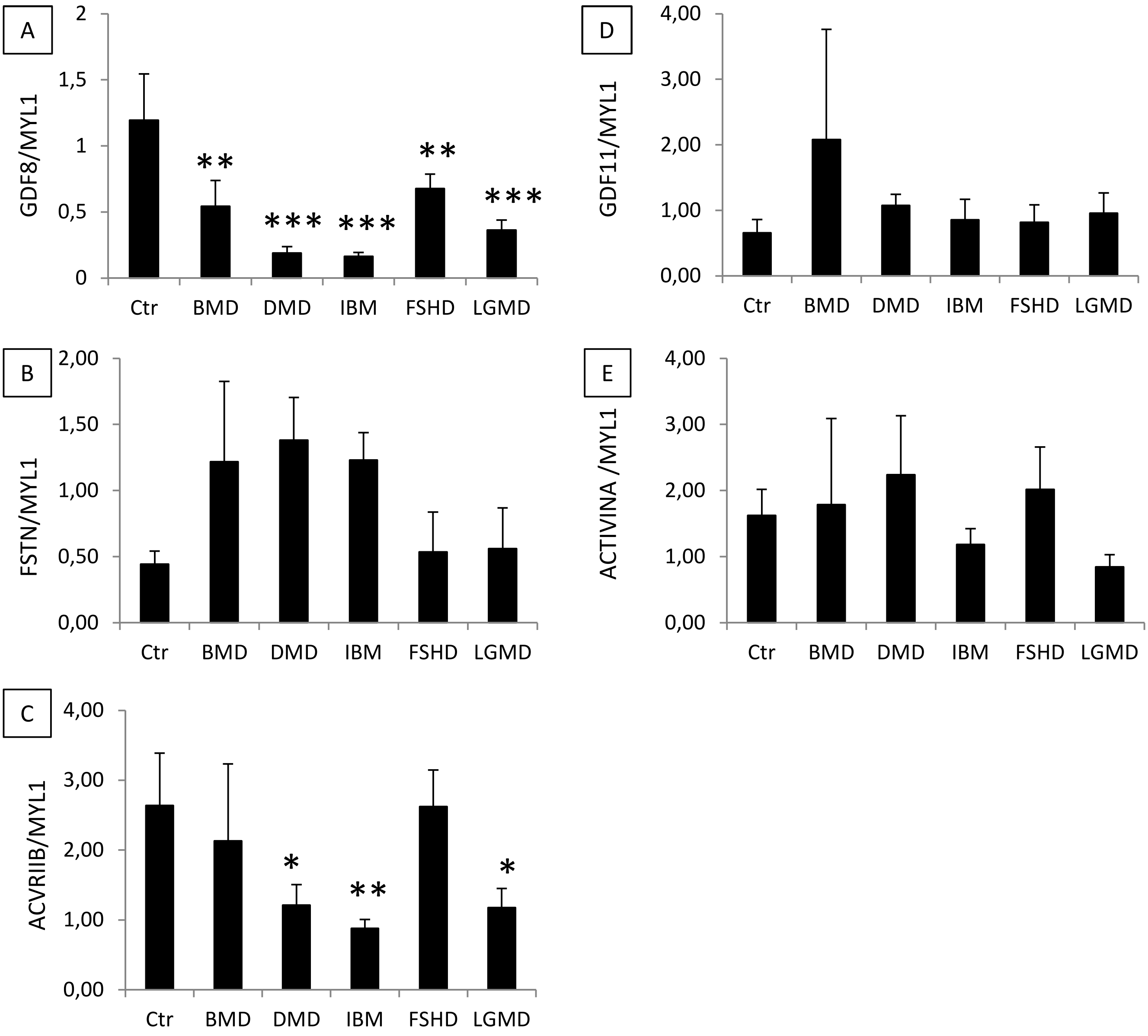


**Supplementary figure 2:** mRNA levels of either *MSTN (A), FSTN (B), ACVRIIB (C), GDF11 (D)* and *ACTIVIN A (E)* were measured by RT-qPCR and normalized by the expression of the MLC-3fast myosin heavy chain (*MYL1*) , in healthy controls (Ctrl, N=9), BMD (N=6), DMD (N=17), IBM (N=17), FSHD (N=13) and LGMD (N=11) patients. All graphs represent mean ± SD. A one-way ANOVA, followed by the Fisher's Least Significant Difference multiple comparison test was performed. p *< 0.05; **<0.01; ***<0.001

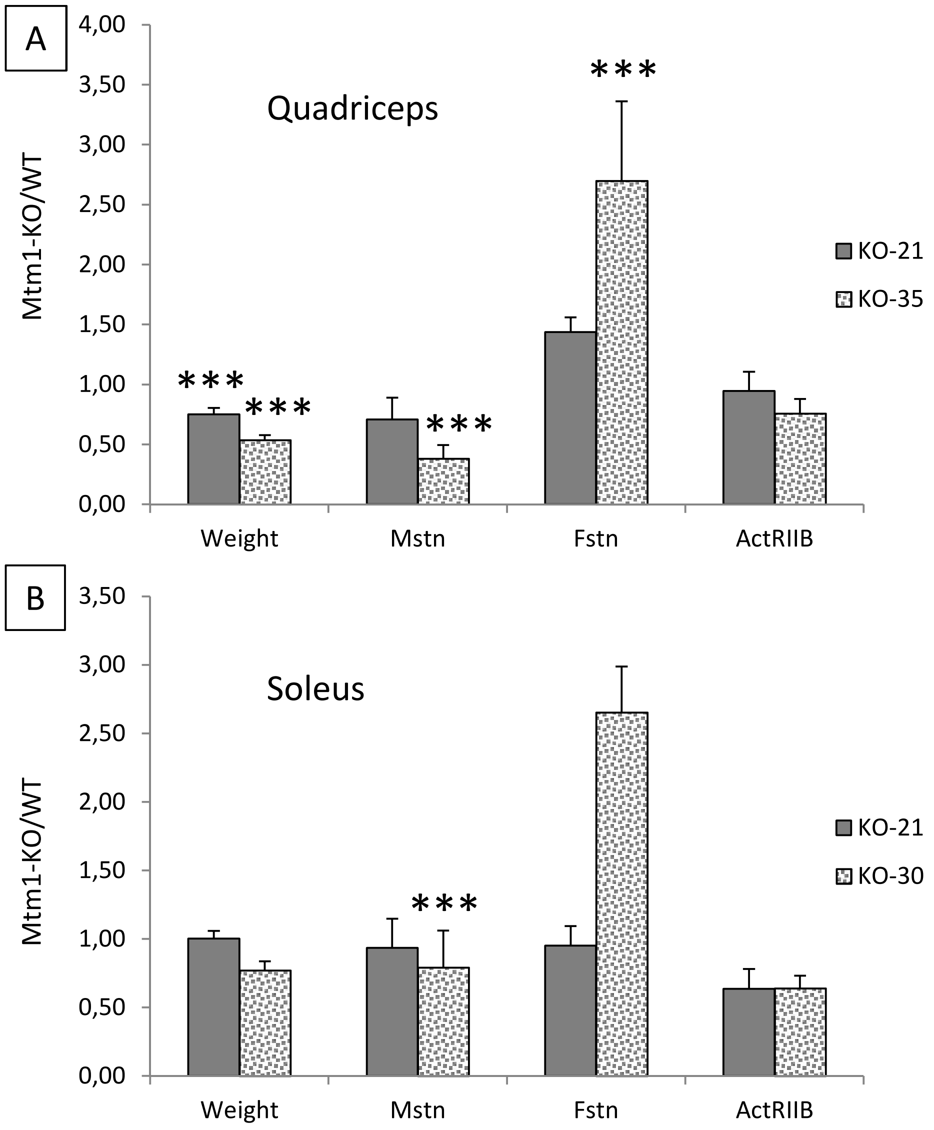


**Supplementary figure 3:** weigth and mRNA levels of either *Mstn, Fstn, AcvrIIb* were measured by RT-qPCR in the quadriceps (A) or the soleus (B) of either 21, 30 or 25 day old Mtm1-KO mice.. n, number of mice used for each analysis: for the quadriceps, n = 9 and 8 at day 21, 8 and 8 at day 35 for WT and KO-Mtm1 mice respectively; for the soleus, n = 8 and 7 at day 21, 9 and 9 at day 35 for WT and KO-Mtm1 mice respectively. All graphs represent mean ± SD. A one-way ANOVA, followed by the Fisher's Least Significant Difference multiple comparison test was performed. p *< 0.05; **<0.01; ***<0.001

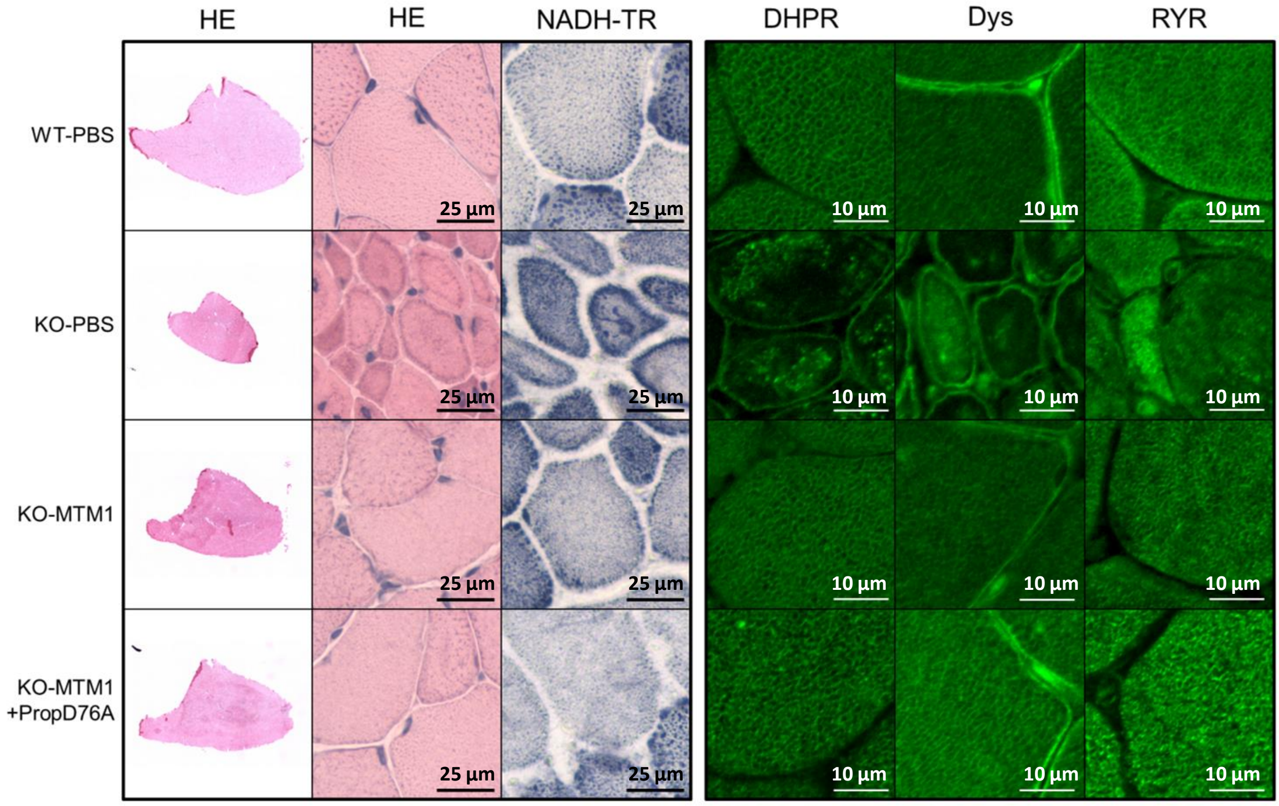


**Supplementary figure 4:** Effect of Mtm1-rescue and mSeAP-PropD76A co-injection on muscle morphological features after 2 weeks of rAAV transduction in the Tibialis anterior (TA) in knockout mouse model. Histological aspect of PBS-treated WT and KO muscle and after injection of a rAAV2/1-Des-Mtm1 and rAAV2/1-CMV-mSeAP-PropD76A. Musclular fiber of TA cross-sections were stained on left panel with hematoxilin and eosin (HE) (magnification × 40 and × 100), mitochondria and endoplasmic reticulum were stained with nicotimanide adenine dinucleotide tetrazolium reductase (NADH-TR) (magnification × 100), and on right panel dihydropyridine receptor (DHPR), dysferlin (Dys) and ryanodine receptor (RYR) were detected by immunofluorescence (magnification × 1200).
